# Control of Nanoscale *In Situ* Protein Unfolding Defines Network Architecture and Mechanics of Protein Hydrogels

**DOI:** 10.1021/acsnano.1c00353

**Published:** 2021-07-02

**Authors:** Matt D. G. Hughes, Benjamin S. Hanson, Sophie Cussons, Najet Mahmoudi, David J. Brockwell, Lorna Dougan

**Affiliations:** 1 School of Physics and Astronomy, Faculty of Engineering and Physical Sciences, University of Leeds, Leeds LS2 9JT, U.K.; 2 Astbury Centre for Structural Molecular Biology, University of Leeds, Leeds LS2 9JT, U.K.; 3 School of Molecular and Cellular Biology, Faculty of Biological Sciences, University of Leeds, Leeds LS2 9JT, U.K.; 4 ISIS Neutron and Muon Spallation Source, STFC Rutherford Appleton Laboratory, Oxfordshire OX11 0QX, U.K.

**Keywords:** protein hydrogels, protein unfolding, hierarchical biomechanics, biomaterials, biomimetic and bioinspired materials

## Abstract

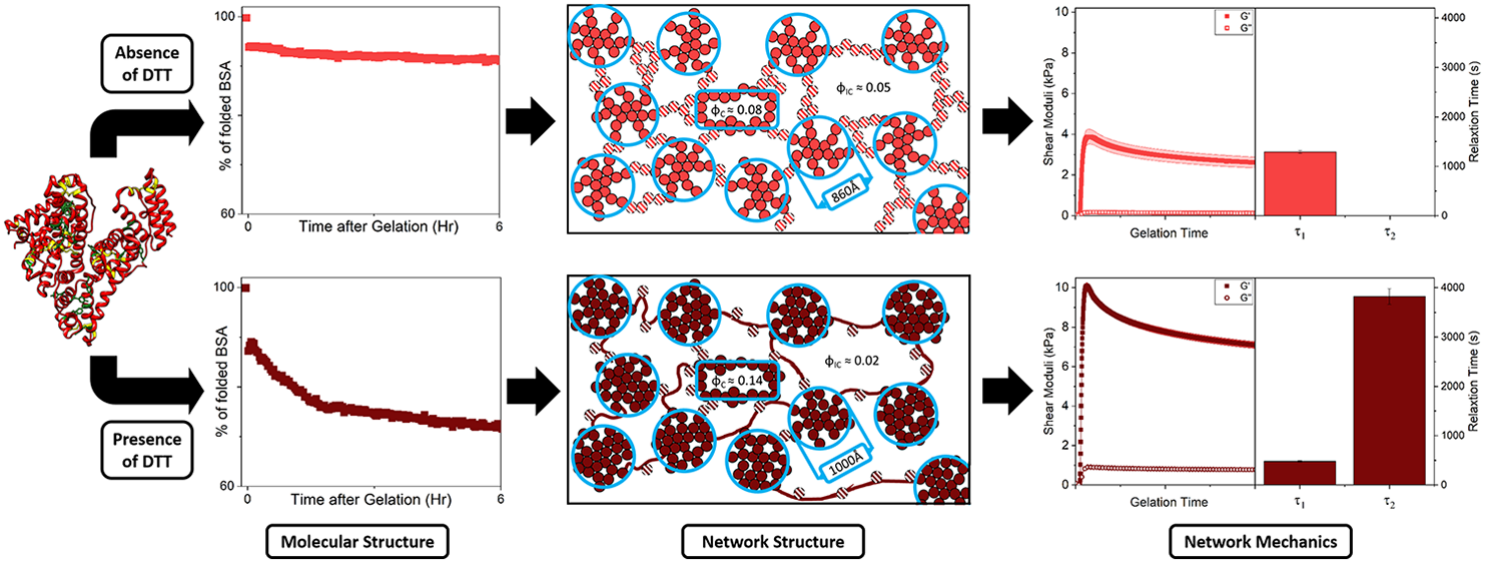

Hierarchical assemblies of proteins exhibit a wide-range of material properties that are exploited both in nature and by artificially by humankind. However, little is understood about the importance of protein unfolding on the network assembly, severely limiting opportunities to utilize this nanoscale transition in the development of biomimetic and bioinspired materials. Here we control the force lability of a single protein building block, bovine serum albumin (BSA), and demonstrate that protein unfolding plays a critical role in defining the architecture and mechanics of a photochemically cross-linked native protein network. The internal nanoscale structure of BSA contains “molecular reinforcement” in the form of 17 covalent disulphide “nanostaples”, preventing force-induced unfolding. Upon addition of reducing agents, these nanostaples are broken rendering the protein force labile. Employing a combination of circular dichroism (CD) spectroscopy, small-angle scattering (SAS), rheology, and modeling, we show that stapled protein forms reasonably homogeneous networks of cross-linked fractal-like clusters connected by an intercluster region of folded protein. Conversely, *in situ* protein unfolding results in more heterogeneous networks of denser fractal-like clusters connected by an intercluster region populated by unfolded protein. In addition, gelation-induced protein unfolding and cross-linking in the intercluster region changes the hydrogel mechanics, as measured by a 3-fold enhancement of the storage modulus, an increase in both the loss ratio and energy dissipation, and markedly different relaxation behavior. By controlling the protein’s ability to unfold through nanoscale (un)stapling, we demonstrate the importance of *in situ* unfolding in defining both network architecture and mechanics, providing insight into fundamental hierarchical mechanics and a route to tune biomaterials for future applications.

It remains a fundamental challenge to relate the properties of an individual nanoscale polymer building block to the collective macroscale response of a network of such building blocks.^1^ The hierarchical structures present in some biopolymer assemblies are crucial to the translation of properties across length scales in living systems^2−4^ and lead to a diverse range of behavior including reversible softening under compression^5^ and both stiffening^6^ and negative normal stress under shear.^7^ New insight would both further our understanding of biopolymer assemblies ubiquitous in living systems and allow for the development of biomimetic and bioinspired materials.^8−11^ Recently, networks of folded globular proteins have been demonstrated to exhibit exciting cross length-scale properties,^12−15^ emerging due to the added complexity and functionality of the folded building block. Since the initial demonstration of folded globular proteins as suitable network building blocks,^15^ protein-based hydrogels have emerged as a new class of biomaterial, exhibiting rich properties such as mimicking the mechanical properties of tissues,^15,16^ forming highly elastic and stimuli-responsive materials,^13,17,18^ and dynamically regulating their properties and shape.^14,19,20^ However, a complete understanding of the translation of nanoscale properties to macroscale networks, which will allow for the rational design of hydrogels with predictable and tuneable properties, remains a fundamental challenge. In this work we demonstrate the potential of the use of nanoscale staples within proteins to define the network architecture and subsequent mechanical response. We will show that manipulation of intraprotein nanostaples provides control of protein mechanics and *in situ* protein unfolding, which is critical for the formation of the precise structure and mechanics of the hydrogel network.

Hydrogels are hydroscopic networks formed from hydrophilic building blocks swollen by relatively large volumes of water, and have become a popular engineered biomaterial, as their high biocompatibility makes them suitable for biomedical applications.^21^ In order to design hydrogels for a specific purpose, it is necessary to understand how to tune and tailor the mechanical properties of the hydrogel to its application. The mechanical properties of hydrogel network structures have previously been modulated using different methods. One approach uses so-called “fillers” to occupy the void space between the connected building blocks in the hydrogel network, restricting movement of the overall network.^22,23^ This approach has resulted in increases in the storage modulus from 6-fold^23^ to 10-fold,^24^ as well as emergent shear stiffening behavior.^25^ An alternative method of hydrogel mechanical reinforcement involves the inclusion of a secondary network in the hydrogel (either permanent^26^ or stimuli-responsive^27^) to act as a scaffold for the original network. These “double-network” hydrogels^28,29^ can be carefully designed to create networks which are “interwoven”, resulting in gels with hybrid mechanical properties^30,31^ such as high mechanical stability of the rigid network and the repetitive elasticity of the flexible network.

Both the filler method and the double network method reinforce and enhance the mechanical properties of the hydrogel network, but both involve the alteration of the hydrogel at the network level rather than on the nanoscale. In contrast, unstructured peptides or those that form α-helices and β-strands have been utilized to investigate how the interpeptide interactions of the network at the molecular level affect the self-assembly of the fibrous microstructure and subsequent mechanics of the hydrogel. Multiple investigations have focused on tuning the structure and mechanics of the hydrogels through precise control of the amino acid composition of these structurally simple, short peptide chain building blocks.^32−35^ These studies and others have demonstrated that changing the hydrophobic interactions of the peptide building blocks, can significantly shift the network morphology, increase the storage modulus by 10–100-fold,^33,34^ and result in interesting thermomechanical properties.^35^

The recent studies on peptide-based hydrogels highlight the critical role of intermolecular interactions on the structure and mechanics of the hydrogel. However, the use of peptides as hydrogel building blocks significantly limits the range of biological functionality. In the past decade, folded globular proteins have been utilized as hydrogel building blocks due to their evolutionarily optimized and highly specialized molecular functions, well defined structures, and thermodynamic/mechanical stability.^36−38^ The folded structures of globular proteins, which display a range of mechanical strengths, provide the opportunity to investigate the translation of mechanical stability of individual building blocks to assemblies of building blocks, all while retaining the inherent biological functionality of the protein. While in-depth analyses of peptide hydrogels using combined multi-experimental technique approaches are common in the literature, in particular through the use of structural techniques in conjunction with bulk mechanical characterization,^33,34,39^ the same detail is lacking in folded globular protein hydrogels.

The additional functionality offered by the mechanically robust well-defined folded structures of proteins offers powerful opportunities for new biomaterials. Recently several studies have studies have (i) examined the interplay between the mechanical stability of the folded protein building block and of the cross-linker;^40^ (ii) investigated the role of rigidity and flexibility in hydrogels by exploiting protein engineering to make constructs containing both folded proteins and unstructured peptide chains, as approximations of rigid rods and flexible chains respectively;^41^ and (iii) determined that the increase of thermodynamic and mechanical stability at the molecular level translates to increased mechanical strength at the network level.^12,14^ All of these studies exploit the nanoscale native state mechanics of globular proteins. However, the importance of the nanoscale transition from a rigid folded to a flexible unfolded state has yet to be explored.

Here, we demonstrate that a transition in the nanoscale structure of the building block *via* protein unfolding has a defining role on network architecture and mechanics. In this work, we present a combined experimental and modeling approach to show that *in situ* unfolding of bovine serum albumin (BSA) modulates the BSA hydrogel network architecture, in particular the intercluster region which demonstrably dominates the mechanical response of the hydrogel network.

## Results

**Figure 1:**
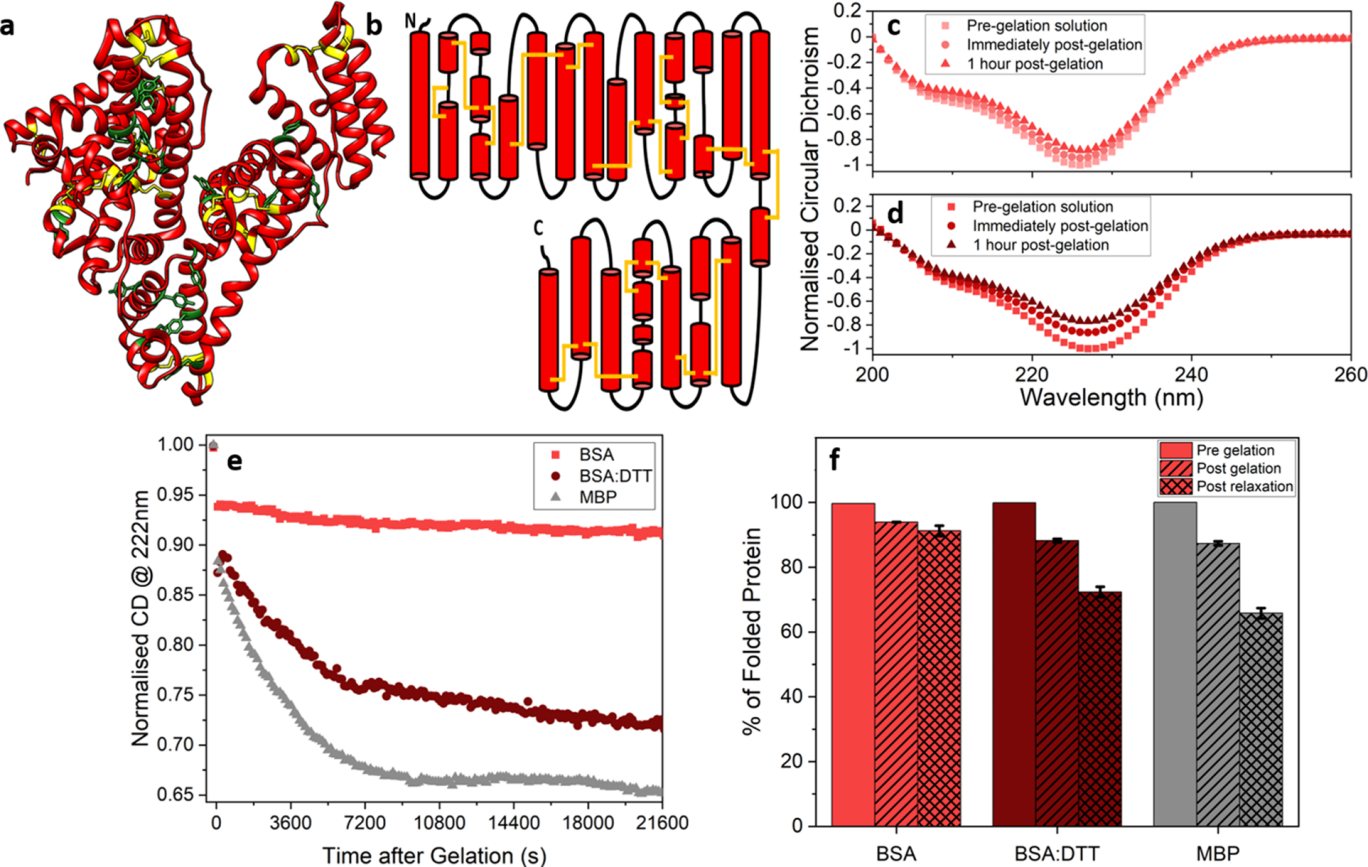
(a) Crystal structures (PDB code: 3v03) and (b) 2D topographs of BSA, where disulphide bonded cysteine residues and tyrosine residues are colored yellow and green, respectively. Normalized circular dichroism spectra of BSA hydrogels in the (c) absence and (d) presence of DTT, before gelation, immediately post gelation and one-hour post gelation. (e) Normalized CD signal at 222nm of BSA (light red) and BSA:DTT (dark red) hydrogels as a function of time post gelation, with previously published data on a hydrogel composed of another globular protein MBP (gray) added for reference. (f) Percentage of folded protein remaining in each hydrogel system, pre-gelation (open), post-gelation (striped), and post-relaxation (cross-hatched). Error bars taken from exponential fit to the curves in part e.

BSA was selected as a model protein to investigate the effects of unfolding of the protein building block *in situ* on the properties of a cross-linked hydrogel network. BSA is an ideal model globular protein as it contains at least four solvent exposed tyrosine cross-linking residues (necessary for the formation of gel network *via* photochemical cross-linking^42^) and, importantly, 17 structural disulphide bonds. These intramolecular disulphide bonds effectively act as “staples” holding the folded structure together (Figure 1a, b) and are prevalent in many protein families that function in an extracellular environment including serum albumins, defensins, and insulins. The covalent staples are mechanically robust and capable of withstanding forces up to 2nN,^43,44^ which is greatly in excess of the 20–100 pN thought to be generated in the cross-linked protein network,^12,45^ yet these bonds are rapidly removed by reducing agents such as dithiothreitol (DTT) used in this study. BSA has disulphide bonds throughout its structure (Figure 1a and b) suggesting that it is highly resistant to force-induced unfolding; however, in the presence of DTT these staples (*i.e.*, the intramolecular disulphide bonds that allow BSA to resist force induced unfolding) break rendering the protein force labile (*i.e.*, readily unfolds under the application of forces present in protein-based hydrogels during gelation). BSA therefore presents the opportunity to determine the importance of force lability of the protein building block on the hydrogel network structure.

In order to confirm the rationale of our selection, we employed circular dichroism (CD) spectroscopy to investigate the structure of the BSA protein in the presence and absence of disulphide bonds both in solution and in the hydrogel.

Figures 1c and d show the spectra of BSA hydrogels in the absence and presence of DTT, respectively, in the pre-gelation solution, in the hydrogel immediately post-gelation and one hour after gelation. From these spectra, a reduction in the protein’s CD signal at 222 nm is observed pre- to post-gelation, which can be interpreted as a decrease in the amount of folded protein in the hydrogel network (α-helix secondary structure in this case) and from which a proportion of folded protein can be extracted. The CD solution spectra (Figure S1a) and the solution structure from small-angle X-ray scattering (SAXS) profiles (Figure S1b) of BSA in the presence and absence of DTT show no significant differences. This demonstrates that the increase in BSA unfolding only occurs in the presence of DTT and internal stress due to gelation. A time course of the CD signal at 222 nm measured *in situ* for hydrogel maturation over 6 h (Figure 1e) shows that the proportion of folded protein in each hydrogel decays over time, approaching end point values after 6 h (Materials and Methods). For BSA, there is a striking difference between the proportion of folded protein in the absence and presence of DTT. While in both chemical conditions there is a decrease in the amount of folded protein after gelation, the extent and rate of decrease is far greater in the presence of DTT (exponential fits to the curves in Figure 1e extract end point values of 9% and 28% in the absence and presence of DTT, respectively). This result implies that removal of these structural staples increases the extent of the gelation-induced unfolding, consistent with the view that structural disulphide bonds provide molecular reinforcement in the BSA folded structure. In accordance with this hypothesis, another folded globular protein that lacks any disulphide bonds^46^ and so is also labile to force (MBP^47,48^) showed behavior similar to disulphide-reduced, unstapled BSA (Figure 1e,f). These results show that the intramolecular disulphide bonds act as molecular staples, reinforcing the BSA building block against force-induced unfolding due to gelation, which leads to the low degree of unfolding observed *in situ*. In contrast, the unstapled BSA in the presence of DTT shows a higher degree of unfolding *in situ* due to their force labile structures. Comparison of these three systems demonstrates that force induced unfolding occurs as a consequence of gelation.

To investigate whether gelation-induced unfolding affected the structure of the cross-linked BSA network, we used small-angle scattering of neutrons (SANS) and X-rays (SAXS). These techniques provide structural information over the length scales of tens to hundreds of Ångstroms.

**Figure 2:**
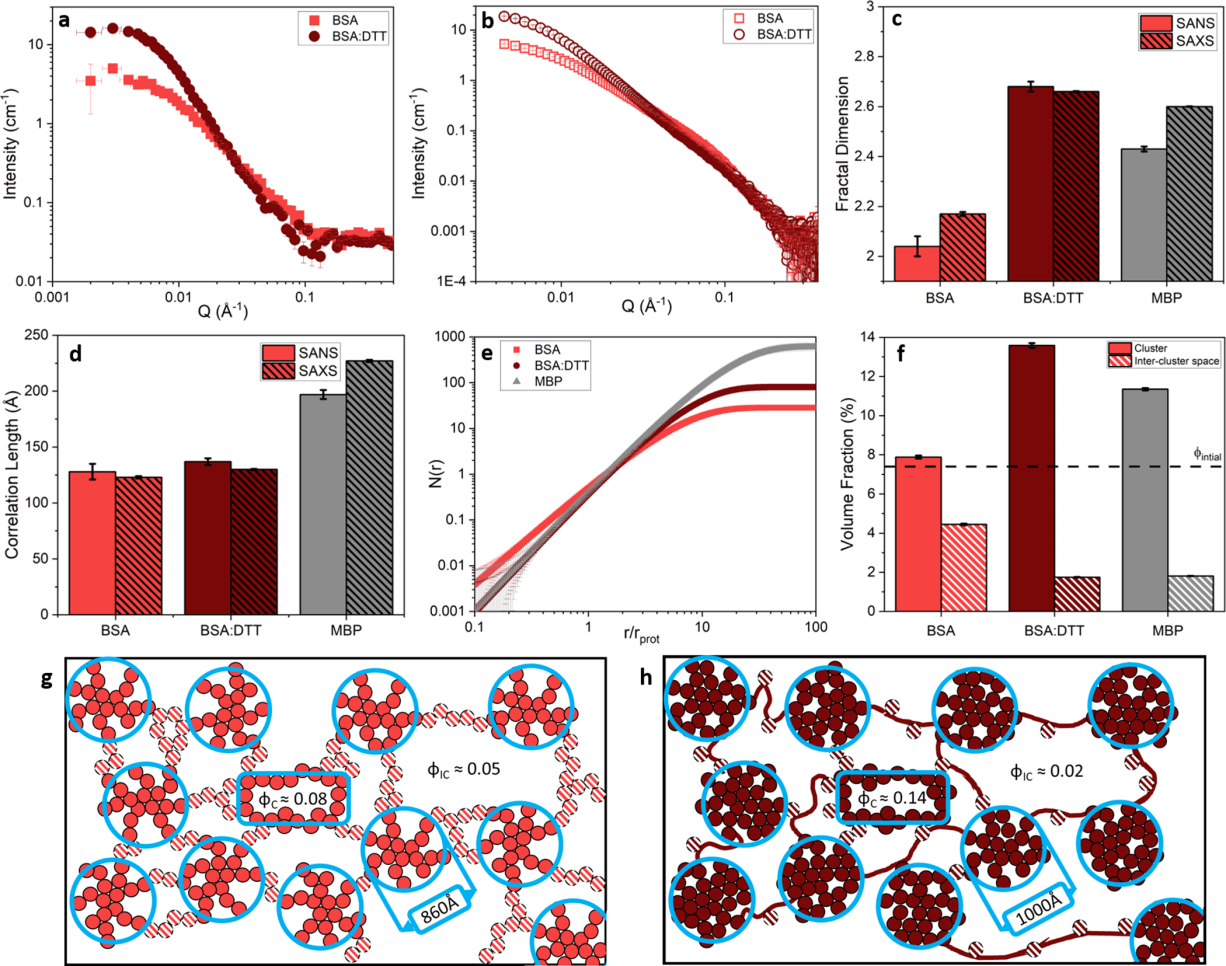
(a) SANS curves and (b) SAXS curves of folded BSA hydrogels (final concentrations: 100 mg/mL BSA, 50 mM NaPS, 100 μM Ru(BiPy)_3_) in the absence (light red) and presence (dark red) of DTT. (c) Fractal dimension and (d) correlation length of clusters present in BSA hydrogels (in the absence and presence of DTT) and MBP hydrogels. (e) Number of protein monomers in a cluster as a function of distance from the center of the cluster. (f) Volume fraction of a cluster (solid color) and intercluster region (red and white striped) for each hydrogel system. Line added at 7.4% to denote the initial volume fraction of the system pre-gelation, ϕ_initial_. (g, h) Schematic representation of the predicted structures of the BSA hydrogel networks in the absence (light red) and presence (dark red) of DTT. Networks consist of cross-linked fractal-like clusters with a volume fraction of ϕ_C_ (represented by solid circles and highlighted by light blue rings) connected by an inter-cluster region of protein with a volume fraction of φ_IC_ (represented by white striped circles). Solid dark red lines represent unfolded BSA protein strands in the intercluster region in the presence of DTT. Error bars show the standard errors, where number of repeats, *N* = 3.

The SANS and SAXS curves of the BSA hydrogels in the absence and presence of DTT are shown in Figures 2a and b, respectively. A qualitative assessment of the scattering curves suggests there are significant structural differences between BSA hydrogels in the absence and presence of DTT, as shown by the reduced intensity at low q values and the shallower slope in the mid *q* range. Interestingly, the BSA hydrogels with DTT show markedly similar profiles to MBP hydrogels (Figure S2). Previous SAS characterization of folded globular protein hydrogels of MBP has shown the presence of discrete fractal-like clusters of cross-linked folded protein in the network structure.^12^ With this model in mind we use eq 1 to extract quantitative information from the curves in Figures 2a and b:
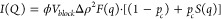
1where ϕ is the volume fraction of protein, *V*_block_ is the volume of the protein building block, Δρ is the contrast difference between the building block and the solvent, *F*(*q*) is the ellipsoidal form factor of the building block, *p*_*c*_ is the proportion of protein in fractal-like clusters within the gel network, and *S*(*q*) is a fractal structure factor.^12^ Two fitting parameters are of core interest in this work, the fractal dimension, *D*_f_, and the correlation length, ξ (see Materials and Methods), values which are displayed in Figures 2c and d. *D*_f_ can be defined as the space-filling capacity of a fractal object which can (and often does) differ from the dimension of the topological space in which the object is embedded. *D*_f_ can also be interpreted as the measure of how the structural detail in an object changes with the scale at which the object is measured.^49^ Sometimes called the mass fractal dimension, it gives a measure of how the “mass” (an intrinsic property) of an object scales with its size (an extrinsic property). For example, if the size of the object increased by a factor of 2, then the “mass” of the object would increase by a factor 2^*D*^f^^. For our system *D*_f_ can be thought of intuitively as related to the density of the clusters of cross-linked folded protein. ξ, then, is an imposed parameter representing the upper limit length scale over which *D*_f_ is a valid measure of hierarchical structure. We interpret this as indicative of the size of the fractal clusters within the network, with the associated lower limit of fractal behavior being the size of an individual protein building block.

The results in Figure 2c show that the measured fractal dimension of a cross-linked cluster is significantly larger in BSA hydrogels formed in the presence of DTT (*D*_f_ = 2.66 ± 0.01 and 2.17 ± 0.01 in the presence and absence of DTT, respectively). While the correlation length also increases in the presence of DTT (ξ = 130 ± 1 Å and 123 ± 1 Å in the presence and absence of DTT, respectively), it is not a significant increase (Figure 2d). This suggests that hydrogels made from force-labile reduced BSA form “denser” fractal-like clusters of a slightly larger size compared to the relatively “sparser” clusters present in hydrogels in the absence of DTT. These results suggest that while the cluster size is unchanged with and without molecular reinforcement, the dimensionality or “density” of the clusters is increased in BSA hydrogels in the presence of DTT. Previously characterized hydrogels constructed from MBP (which has no disulphide bonds) are shown for reference^12^ and are again similar to the BSA hydrogels in the presence of DTT, exhibiting clusters of protein with similar dimensionality (*D*_f_ = 2.6 ± 0.01) but larger cluster size. MBP hydrogels were the same volume fraction (ϕ = 7.4%) as the BSA hydrogels (ϕ = 7.4%) presented in this work, but due to the difference in protein size (*r*_MBP_ ∼ 24 Å vs *r*_BSA_ ∼ 33 Å) a greater number of MBP monomers are required to make up the same volume fraction. This difference in number of monomers results in larger clusters containing more protein in MBP hydrogels compared to BSA:DTT hydrogels, though the network topology remains the same.

To gain more insight into the hydrogel cluster size and morphology, we consider the radial distribution function, *g*(*r*), determined by Teixeira^50,51^ to derive the fractal structure factor (eq 2):
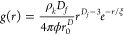
2where ρ_*k*_ is the maximum packing density of the system, *i.e*. for randomly assembled spheres 0.637, and *r*_0_ is minimum cutoff distance of the fractal cluster, *i.e*. the effective radius of the building block. The exponential term is introduced with the parameter ξ to act as a cutoff distance, imposing a maximum size on the fractal cluster, as discussed earlier.^50^ Multiplying the radial distribution function by the volume fraction of the system and integrating over *r* gives an expression for the number of individual building blocks in a sphere of radius, *r*, from the center of the cluster (eq 3):
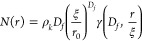
3where γ(*D*_*f*_, *R*/ξ) is the lower incomplete gamma function. Figure 2e shows how the number of protein building blocks varies as a function of distance from the center of a fractal-like cluster in units of the building block radius. In all cases the curves increase at a rate related to the fractal dimension of the cluster and plateau at large distances from the cluster center. This is expected given the exponential term in eq 2, giving a measure of the maximum number of building blocks in a cluster. We find that BSA hydrogels have approximately 7 times more protein in each cluster in the presence of DTT, suggesting an extremely important role of protein unfolding in determining the cross-linked cluster density. From the calculated curves in Figure 2e we can extract an estimate of the radius of the fractal-like clusters (Figure S3). The increase in the cluster size in the presence of DTT is consistent with the same increase observed in the correlation length, which is to be expected as the distance at which *N*(*r*) plateaus (Figure 2e) is completely dependent upon the correlation length. Using an estimate of the cluster size and proportion of protein in the fractal-like clusters, we can calculate the volume fraction of a fractal-like cluster in isolation (φ_C_) and the associated intercluster region (φ_IC_) in the hydrogel (Figure 2f). In both the absence and presence of DTT, φ_C_ is larger than φ_IC_, suggesting a heterogeneous hydrogel network dominated by clusters of proteins. The volume fractions of clusters and intercluster regions change upon addition of DTT, with denser clusters and a sparser intercluster region, suggesting a more heterogeneous network. Interestingly, the clusters formed in the absence of DTT have a volume fraction very close to the initial volume fraction pregel solution (7.89 ± 0.08% versus 7.4%). This result is consistent with what is expected from diffusion-limited cluster aggregation theory,^52,53^ in which individual particles undergoing Brownian motion aggregate together to form clusters of such particles. A consequence of this theory is that clusters will continue to grow in size, until their volume fraction is equal to the initial volume fraction of the solution. This result implies that the predominate mechanism in the formation of BSA hydrogels in the absence of DTT is diffusion-limited cluster aggregation, as opposed to reaction-limited aggregation.^54,55^ In the presence of DTT, however, the volume fraction of BSA clusters (13.6 ± 0.1%) differs significantly from the initial pregel volume fraction, suggesting an additional mechanism involved in the formation of these hydrogels. For reference, this analysis was also performed on previously published SAS data on MBP, included in Figures 2c–f, showing large levels of heterogeneity in the network comparable to that of BSA hydrogels in the presence of DTT. All the data therefore suggests that both BSA in the presence of DTT and native MBP will yield and unfold to applied force, whereas native stapled BSA does not yield to applied force.

Combining the force lability of the protein building block and CD and SAS structural analysis, we propose a model of the network structure of folded globular protein hydrogels, shown in Figure 2g and h. Folded proteins with covalent intramolecular disulphide bonds are unyielding to force and form hydrogel networks with fractal-like clusters made up of proteins connected by intermolecular dityrosine cross-links, with clusters linked together by multiple folded proteins (Figure 2g). Breakage of the intramolecular disulphide bonds yields a force labile protein, and denser fractal-like clusters are formed, with clusters connected by unfolded protein (Figure 2h). The force lability of the protein is crucial in modulating the structure of the hydrogel networks.

In order to gain insight into the evolution of the structure from monodispersed solution to a self-supported network, we employ a previously used dynamic computational model: BioNet.^56,57^ BioNet can model individual folded protein monomers by representing them as freely diffusing and rotating, pseudo-deformable (soft-core potential) spheres with explicit cross-linking sites defined at the sphere surface. When within 3 Å of one another, a rigid bond will form between these sites to represent the cross-linking mechanism. To approximately model the BSA subunit, each sphere was given a radius of 33 Å with 14 evenly spaced cross-linking sites defined (representing the tyrosine residues in BSA) and another 4 randomly placed in the remaining space. These spheres then undergo a Brownian dynamics protocol with a local drag on each sphere. We are also able to model unfolded BSA as a chain of interacting binding sites connected by Hookean springs. These Hookean springs represent the end-to-end fluctuations expected of the worm-like chain polymer model, specifically where the length of the overall polymer is significantly greater than its persistence length as is the case for fully unfolded protein. To ensure the relative diffusion time scales of the unfolded protein components were appropriate, we assigned a local drag to each point-like binding site to approximately match the drag on each segment of the amino-acid chain between binding sites.

We consider two key cases: one where the simulation is initialized with 91% of the monomers in a “folded” state and one initialized where 72% of the monomers are in the “folded” state. These were chosen as close approximations of BSA hydrogel in the absence and presence of DTT, respectively (Figure 1f). The simulations were robustly initialized with periodic boundary conditions applied and continued until the networks were sufficiently percolated (Materials and Methods). We emphasize that in these simulations, the explicit unfolding of protein monomers during the “gelation” process is not modeled, as protein unfolding is highly non-trivial due to the complexity of the pulling direction^58^ and also the effects of crowding.^59^

**Figure 3:**
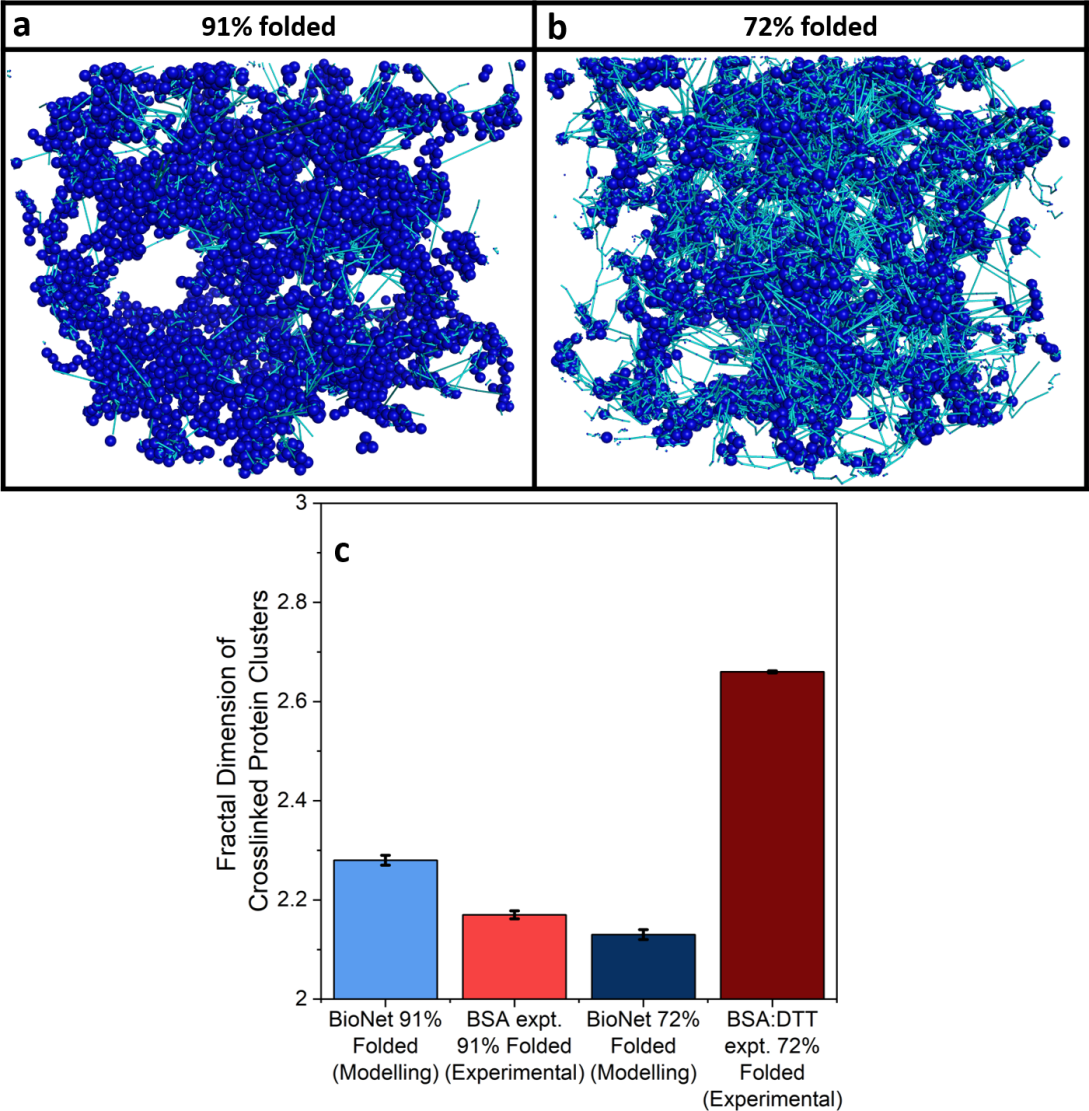
(a, b) Schematics representing the explicit structures calculated using BioNet simulations, where the blue spheres represent folded protein and the lines represent connections made by unfolded protein. (c) Fractal dimensions of cross-linked clusters extracted from both BioNet simulations of a “single cluster” (calculated using a box counting method) and experimental SAS data (Figure 2). The proportion of unfolded protein for the experimental results is the post-relaxation end point values taken from Figure 1e. In contrast, for the simulations the proportions of folded protein represent the fixed amount of unfolded present in the simulation box over the course of the simulation. Error bars show the standard errors, where number of repeats, *N* = 3.

Once the simulations are complete, a box counting method was employed to extract an explicit value for the fractal dimension from each of the simulated cross-linked clusters (Materials and Methods, see Figure S4). The fractal dimensions extracted from both the simulations and experimental data are shown in Figure 3. The fractal dimension extracted from the simulation with 91% folded monomers (2.28 ± 0.01) is in reasonably good agreement with experimentally measured BSA hydrogels in the absence of DTT (2.16 ± 0.01). However, the fractal dimension extracted from simulations containing only 72% folded monomers (2.13 ± 0.01) is significantly different to our experimental results of BSA hydrogel in the presence of DTT (2.66 ± 0.01). However, previous BioNet simulations of monodisperse systems of 100% folded proteins (i.e. spheres only) showed that a lower fractal dimension is to be expected for systems at lower volume fractions56, showing that the homogeneous presence of mechanically weak polymeric chains effectively acts as almost empty space with respect to the fractal dimension. Hence, comparison of the simulation and experimental results (Figure 3) indicates that a general, homogeneous presence of unfolded protein throughout the system during gelation is not sufficient to cause the large structural changes in the hydrogel architecture observed in our experimental SAS data (Figure 2). The combination of experimental and computational results therefore suggests that it is the act of unfolding of specific force labile protein building blocks during gelation itself that is crucial in defining the hydrogel architecture. However, it is possible that the change in hydrogel network structure observed experimentally involves aggregation of the unfolded protein chains, which is not captured by our simulation. To confirm our hypothesis that it is the act of unfolding that defines hydrogel architecture, further investigation beyond the scope of this work would be required, including rapid frame acquisition SAXS and computational modeling that accurately models dynamic unfolding during gelation and the behavior of unfolded protein chains.

The results above show that removal of disulphide cross-links within BSA monomers affects the resulting hydrogel structure at the molecular and network level, due to the force-induced unfolding of the BSA building block that occurs in the absence of disulphide “staples”. To investigate the effects of these structural changes on the macroscopic mechanics, we performed rheology experiments on the BSA hydrogels in the absence and presence of DTT.

**Figure 4:**
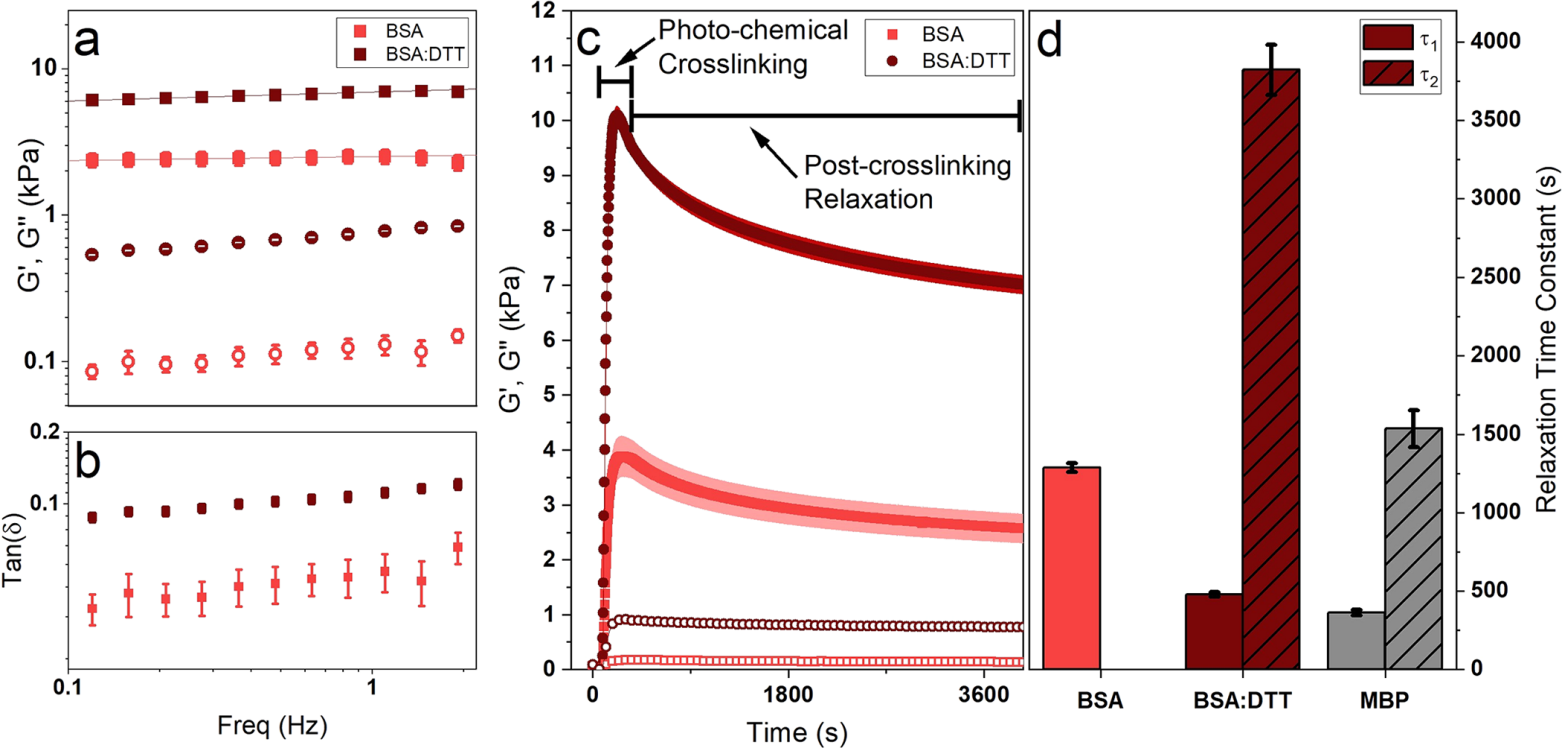
(a) Frequency sweeps showing the (filled) storage, *G*′, and (open) loss moduli, *G*′′, of chemically cross-linked BSA hydrogels (final concentrations: 100 mg/mL BSA, 50 mM NaPS, 100 μM Ru(BiPy)_3_) in the absence and presence of 3 mM DTT. An oscillatory strain of 0.5% was applied to each sample. (b) tan(δ) of BSA hydrogels as a function of applied frequency in the absence and presence of DTT. An oscillatory strain of 0.5% was applied to each sample. (c) Gelation curves, showing storage (closed symbols) and loss moduli (open symbols) vs time of BSA hydrogels in the absence (light red) and presence (dark red) of DTT. Illuminated at *t* = 60 s till *t* = 360 s. (d) Time scales of relaxation modes in BSA hydrogels with MBP hydrogels added for reference. Error bars and ribbons show the standard errors, where number of repeats, *N* = 3.

Figures 4a and b show how the components of the complex shear modulus of BSA hydrogels, *G*′ and *G*′′ (storage and loss moduli, respectively), and the loss ratio, tan(δ) (defined as *G*′′/*G*′), vary with applied oscillatory frequency in the absence and presence of DTT. The storage modulus, which is a measure of the hydrogel elasticity, is approximately 3-fold higher in the presence of DTT, while the loss modulus, which is a measure of the hydrogel viscosity, is approximately 5-fold larger. Fitting a linear function to the storage modulus allows for the extraction of the power law exponent giving an insight into the dynamics of the system (Materials and Methods). The extracted exponent, known as the relaxation exponent, *n*, is a measure of the frequency dependent behavior of the system and for gel-like systems gives information on the relative dominance of elastic or viscous behavior. A gel with *n* approaching 1 is a purely viscous gel whereas a gel with *n* approaching 0 is a purely elastic gel.^60−62^ The *n* values are 0.027 ± 0.002 and 0.061 ± 0.001 in the absence and presence of DTT, respectively. From these values it can be seen that both gels exhibit elastically dominated behavior; however, the increase in exponent in the presence of DTT suggests an increased level of viscosity in the BSA:DTT hydrogel. This increased level of viscosity is likely due to the increased proportion of unfolded protein present in the hydrogel (Figure 1f). This is also consistent with the results in Figure 4b in which tan(δ) is higher in the presence of DTT, showing a higher level of viscosity in BSA hydrogels in the presence of DTT.

In addition to the enhancement of the storage modulus in the presence of DTT, there is also an increase in the viscous behavior of the hydrogel as denoted by an increase in the loss ratio, which is consistent with the increased amount of unfolded protein in the system in the presence of DTT as confirmed by CD (Figure 1c–f). This increase in the storage modulus of BSA hydrogels gelated in the presence of DTT may be due to additional cross-links in the intercluster region, either physical or chemical, between the force unfolded protein chains. To explore this further, a BSA hydrogel formed in the absence of DTT (*i.e.*, fully folded) was soaked *in situ* on the rheometer in a 3 mM DTT solution post-gelation (Figure S5a), which resulted in a decrease in the storage modulus (Figure S5b). This decrease in storage modulus can be attributed to the force induced unfolding of load bearing BSA building blocks as the DTT diffuses into the gel. Upon soaking of the BSA hydrogel in DTT, an increase in the loss ratio (Figure S5c) is noted, demonstrating an increase in the viscous behavior, which is consistent with an increase in the amount of unfolded protein in the gel. This decrease in the storage modulus (and simultaneous increase in the viscous behavior) upon soaking suggests that no additional physical cross-links are being formed between the unfolded protein chains, as we would expect this to increase the value of *G*′. The enhancement to the storage modulus of BSA hydrogels in the presence of DTT (Figure 4a,c) is therefore not due to additional physical cross-links but a result of additional chemical cross-links formed by the force unfolded protein chains during the photochemical gelation process.

To further investigate the molecular reinforcement of the protein and its impact on hydrogel formation, we analyzed the changes in storage (*G*′) and loss (*G*′′) moduli of BSA hydrogels in the absence and presence of DTT during gelation, as a function of time, again using rheology (Figure 4c). In both cases the curves initially show a dramatic increase in *G*′ during the photochemical cross-linking process, which is then followed by a large relaxation to a final value of *G*′. Fitting these curves with a previously used empirical function^12^ (eq 7, Materials and Methods) allows us to extract information on the relaxation behavior of the system. Figure 4d shows the extracted time constants of relaxation of the system. The difference between the relaxation behavior of BSA hydrogels in the absence and presence of DTT is striking, with the former having one mode of relaxation (τ_1_ = (1290 ± 30) s) while the later has two distinct relaxation modes (τ_1_^DTT^ = (480 ± 10) s, τ_2_^DTT^ = (3800 ± 200) s). In a previous study of a folded protein hydrogel using MBP,^12^ we measured two modes of relaxation. A fast relaxation was attributed to the formation of a percolated hydrogel network, and a second, slower relaxation was attributed to the unfolding of the protein building block. Interestingly, BSA in the absence of DTT displays one relaxation mode, while in the presence of DTT we see two (Examplar fits shown in Figure S6). The τ_2_^DTT^ values extracted from the gelation curves in the presence of DTT are similar to the time scale of unfolding observed in CD and in our previous work.^12^ In combination with CD data (Figure 1c–f) this suggests that the emergence of two-relaxation modes is inherently linked to force lability of the protein during gelation. Note, this same behavior is observed for BSA hydrogels which are then soaked in DTT solution (Figure S5a and Figure S5d). Therefore, we have demonstrated that the relaxation behavior of folded protein-based hydrogels is intimately linked to the force lability of the protein building block.

**Figure 5:**
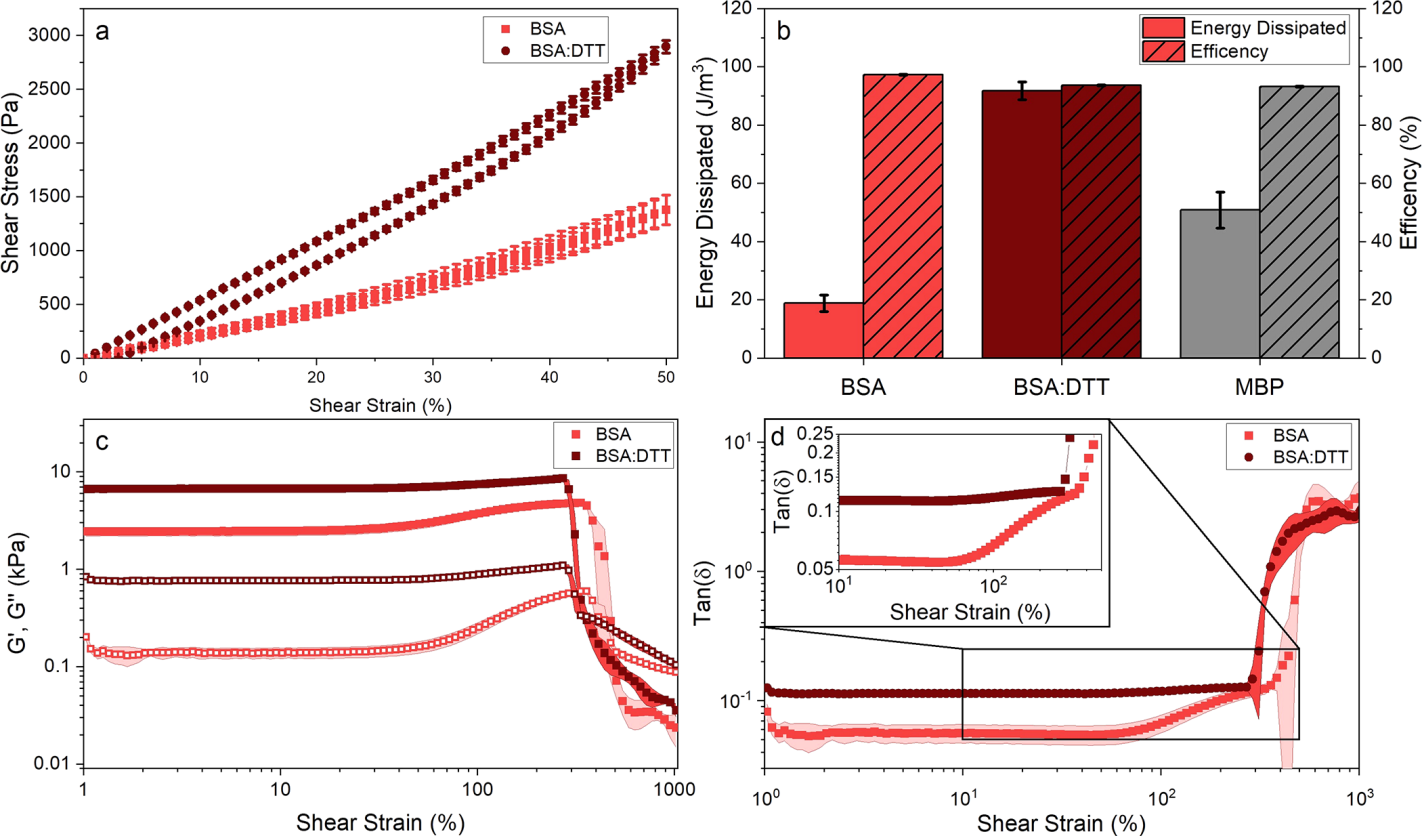
(a) Stress–strain curves of chemically cross-linked BSA hydrogels (final concentrations: 100 mg/mL BSA, 50 mM NaPS, 100 μM Ru(BiPy)_3_) in the absence and presence of 3 mM DTT. (b) Energy dissipation (open) and efficiency (striped) during the load–unload cycle of BSA hydrogels in the absence (light red) and presence (dark red) of DTT. Data on MBP hydrogels (gray) added for reference. (c) Storage and loss moduli and (d) tan(δ) of BSA hydrogel in the absence and presence of DTT as a function of applied oscillation strain at 1 Hz. (inset) Enlargement of the strain-stiffening regime, plotted without error bars for clarity. Error bars and ribbons show the standard errors, where number of repeats, *N* = 3.

In addition to the analysis of the linear mechanics of BSA hydrogels, the behavior of the system under load was explored. Figure 5a shows shear stress–strain loading curves of BSA hydrogels from applied rotational rheology in the absence and presence of DTT. In either condition, the stress–strain curves show linear elasticity at shear strains of less than 25% (Figure 5a). Fitting this linear region yields storage moduli (*G*′) of 2.6 ± 0.3 kPa and 6.3 ± 0.2 kPa (in the absence and presence of DTT, respectively) in good agreement with the values in Figure 4a. The stress strain curves also display hysteresis behavior upon unloading of the sample and are particularly prominent in BSA hydrogels in the presence of DTT. The hysteresis area enclosed by the stress–strain curves is a quantitative measure of the energy dissipated to the internal energy of the system. Integrating the stress–strain curves allows for the extraction and calculation of the energy dissipated and the efficiency of the hydrogels (shown in Figure 5b). In the presence of DTT there is an over 4-fold increase in the energy dissipated, while there is only a 3% reduction in the efficiency of the gels from 97% in the absence and 94% in the presence of DTT. This increase in energy dissipation is likely due to larger amounts of unfolded protein in samples in the presence of DTT, as upon unloading energy is lost to the rearrangement of the unfolded peptide chains. This interpretation is consistent with the CD results (Figure 1c–f), our structural model (Figure 2g,h), and previous literature^13,63^ (which observed greater hysteresis behavior in samples with more unfolded protein).

Finally, the nonlinear mechanical behavior of the BSA hydrogels was investigated; the shear moduli and loss ratio of the hydrogels under increasing strain are shown in Figures 5c and d, respectively. The graphs show a linear trend up to strains of approximately 40%, after which there is a stiffening region until rupture at strains of approximately 300%. Interestingly a far larger degree of strain stiffening is noted in BSA hydrogels in the absence of DTT than those in the presence of DTT. This difference is likely due to the difference in structures (Figure 2g,h), in which the native BSA gels have many folded domains that will act as load bearing molecules as the strain is increased, whereas gels in the presence of DTT have a large proportion of unfolded proteins which uncoil toward their full contour length under strain meaning very little change would be seen in the shear moduli.

## Conclusions

We have demonstrated that control of protein force lability has an important role in defining the architecture and mechanics of cross-linked protein hydrogels. We show that a network made from an internally stapled protein building block retains 91% of its protein in the folded state compared to 72% folded protein in a network made from unstapled protein (Figure 1f). This result implies protein reinforcement reduces the probability of force induced protein unfolding during gelation. The network structure formed from a disulphide-protected folded protein, that is unyielding to force, consists of fractal-like clusters made up of cross-linked protein, with the linking intercluster region populated by folded proteins (Figure 2g). Without molecular reinforcement, the protein building block is force labile and denser fractal-like clusters are formed, with the connecting intercluster region populated by unfolded protein (Figure 2h). By complementing our experimental work with computational modeling (Figure 3), we infer that it is the act of unfolding of specific force labile protein building blocks during gelation, rather then just the presence of unfolded protein, that is crucial in defining the hydrogel architecture. To confirm this hypothesis, further investigation beyond the scope of this work would be needed including rapid frame acquisition SAXS and computational modeling that accurately models unfolding during gelation.

Controlling the force lability of the protein building block also has a significant impact on the mechanics as well as the architecture of the protein hydrogel network. Networks formed from force labile protein exhibit a higher elasticity (storage modulus approximately 3-fold higher), enhanced viscous behavior, and energy dissipation, relative to the networks formed from internally stapled protein (Figures 4 and 5). We suggest that this increase in viscous behavior is due to a higher prevalence of unfolded protein in the intercluster region of the hydrogel network constructed from the force labile protein building block. The increase in the elasticity of the network is attributed to additional chemical cross-links in the intercluster region of the network, formed between the strands of force-induced unfolded protein in this region. These results suggest that controlling the building block unfolding and cross-link density in the intercluster region is key in regulating and defining the mechanics of the network. Interestingly, the dominance of the intercluster region on the mechanical response of a network has been observed by other groups in colloidal systems. Del Gado et al. and Frust et al. have found that the connections between clusters in the intercluster region, termed the “rigidity percolation network”, are key in regulating the mechanics of colloidal networks both theoretically^64,65^ and experimentally.^66^ These studies similarly found that heterogeneity in the network structure was crucial in governing the mechanical response of the network.

Conversely, studies on peptide-based hydrogels have shown that as the network becomes more homogeneous^33,34^ the mechanical strength is enhanced. We speculate that this difference is due to the contrasting structures between the two systems, with interconnected clusters and web-like fibrous structures exhibited by folded protein and peptide gels, respectively.

Restriction of unfolding of the protein building block also has a significant impact on the relaxation behavior of the hydrogels. While a single mode of relaxation describes the networks formed from stapled protein, a dual relaxation mode is necessary for networks formed from the unstapled protein. We propose the additional mode of relaxation in protein networks constructed from unstapled protein corresponds to unfolding of the force labile protein (Figure 4). Additionally, the relaxation mode corresponding to unfolding is observed in hydrogels constructed from a stapled protein soaked in DTT post-gelation (Figure S5) accompanied by an approximate factor of 2 reduction in the storage modulus, consistent with previously published literature^41^ comparing rigid and flexible building blocks.

The modulation of the force lability of the building block plays a fundamental role in defining the network architecture and mechanics. The transition of the building block from a rigid folded state to a flexible unfolded state emerges as a powerful method for controlling the intercluster region of the network structure and the subsequent mechanical response. This knowledge provides a powerful route to be exploited in protein engineering, whereby the force lability of the protein can be manipulated in subtle and specific ways. The single molecule community has provided rich information on protein mechanics and their rational design through approaches including protein engineering, ligand binding, and external stimuli (including pH and temperature) and routes to manipulating the local and global mechanical properties of protein and unfolding.^36,67−71^ TThese studies provide inspiration for the continued development of a tool-box of force labile protein building blocks and their incorporation into protein hydrogels. Furthermore, the results of single molecule force experiments and computational models may be able to act as a direct parallel to the internal behavior of protein hydrogels. This study has demonstrated the necessity of combined structural and mechanical characterization to understand the translation of complex molecular properties across length scales. By understanding the crucial role of building block unfolding on hierarchical networks, we demonstrate the importance of *in situ* unfolding in defining the structural and mechanical behavior of the network and reveal building block unfolding as a method for the design of biomimetic and bioinspired materials.

## Materials and Methods

### Materials

Bovine serum albumin (heat shock fraction, protease free, fatty acid free, and essentially immunoglobulin free), tris(2,2′-bipyridyl)dichlororuthenium(II) hexahydrate (Ru(BiPy)_3_), sodium persulfate (NaPS), 1,4-dithiothreitol (DTT), d-(+)maltose monohydrate, sodium phosphate dibasic, and sodium phosphate monobasic were obtained from Sigma-Aldrich and used without further treatment. *N-*Terminal hexa-histidine tagged MBP was expressed and purified as described below.

### Protein Preparation

For completeness the preparation method of MBP has been included. MBP was prepared using a mutated pMal-c5x vector, with a stop codon inserted at position 378 by Q5 mutagenesis. The mutated vector was transformed into the expression host *Escherichia coli* BL21 (DE3) pLysS competent cells. Selected colonies were grown overnight in Lysogeny Broth (LB) at 37 °C, 200 rpm to form starter cultures. 2 mL of these starter cultures were used to inoculate 0.5 L of autoinduction media^72^ in 2.5 L conical flasks. Cultures were incubated for 48 h at 37 °C, 200 rpm before cells were harvested at 8000 rpm for 45 min. The harvested pellets were resuspended in lysis buffer (0.1% Triton X-100, 1 mM PMSF, 20 mM benzamidine, 20 mM Tris, 300 mM NaCl, 10 mM imidazole, pH 8), homogenized and incubated for 1 h in the presence of DNAase. Cell solutions were passed through a cell disruptor (30 Kpsi, 25 °C), to ensure complete lysis, before centrifuging at 25,000 rpm for 25 min to pellet the cell debris and collect the lysate.

To purify the MBP from the lysate, it was loaded onto a Ni-NTA resin column overnight at 2 mL/min to ensure maximum binding of the hexa-histidine-tagged MBP. The column was then equilibrated in wash buffer (20 mM Tris, 300 mM NaCl, 10 mM imidazole, pH 8), before the protein was eluted with elution buffer (20 mM Tris, 300 mM NaCl, 500 mM imidazole, pH 8) in a ratio of 1:3 to wash buffer. The purified protein was dialyzed into water and freeze-dried for storage at −20 °C. Average MBP yields of 300 mg per liter.

### Sample Preparation

As previously published, hydrogel samples are prepared by mixing in a 1:1 ratio a 200 mg/mL stock of either BSA or MBP protein and 2× cross-link reagent stock for final protein and reagent concentrations of 100 mg/mL BSA (MBP), 50 mM (30 mM) NaPS, and 100 μM Ru(BiPy)_3_.

### Circular Dichroism (CD)

Far-UV circular dichroism spectra of MBP hydrogels were acquired on a Chirascan plus circular dichroism spectrometer (Applied PhotoPhysics) with a bandwidth of 2 nm, a step size of 1 nm, and a commercially available cuvette (Hellma) with a path length of 10 μm. The BSA samples contained either 0 mM or 3 mM DTT. Time-course CD measurements were acquired at 23 °C over a long-time scale (approximately 10 h) to allow for correction to the data due to dehydration. Dehydration was corrected for by fitting the natural log of the data at large *t* (>6 h) to determine the rate of dehydration. This rate was used to fit the whole data set with a double exponential decay function (in which one of the rates was fixed to the rate of dehydration), and the exponential decay term corresponding to the dehydration was removed.

### Small Angle Scattering (SAS)

SAS curves were fitted using SasView (http://www.sasview.org) in accordance with eq 1.
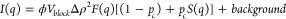
4
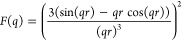
5
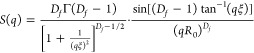
6where *F*(*Q*) is an ellipsoidal form factor^73^ and *S*(*Q*) is a fractal structure factor to model the geometry of the clustering of objects of the form *F*(*Q*).^50^*D*_*f*_, ξ, and R_0_ are defined as the mass fractal dimension, correlation length, and minimum cutoff length scale defined by the ellipsoid form factor, respectively.

### Small Angle Neutron Scattering (SANS)

SANS measurements were conducted on the time-of-flight instrument Sans2d at the ISIS Neutron and Muon Source (STFC Rutherford Appleton Laboratory, Didcot, UK). Sans2d front and rear detectors were set up at 5 and 12 m, respectively, from the sample, defining the accessible *q*-range as 0.002–0.5 Å^–1^. Temperatures were controlled by an external circulating thermal bath. Samples were loaded and gelled in 1 mm path length quartz cuvettes. The raw SANS data were processed using the Mantid framework^74^ following the standard procedures for the instrument (detector efficiencies, measured sample transmissions, absolute scale using the scattering from a standard polymer, *etc.*).^75^

### Small Angle X-ray Scattering (SAXS)

SAXS measurements were conducted in the Materials Characterization Laboratory of the ISIS Neutron and Muon Source, on the Nano-inXider instrument (Xenocs, Sassenage, France) using a microfocus sealed-tube Cu 30 W/30 μm X-ray source (Cu Kα, λ = 1.54 Å). Samples were loaded and gelled in 1 mm path length glass capillary tubes. The *q*-range investigated was 0.0045–0.37 Å^–1^, and measurements were made at room temperature.

### Rheometry

Mechanical characterization experiments of BSA hydrogel samples were performed on a Anton Parr MCR 502 stress controlled rheometer (Anton Parr GmbH, Austria) in parallel plate configuration (with a plate diameter of 8 mm). Photochemical cross-linking was initiated and controlled *via* illumination by blue LED (peak emission at 452 nm) at a current of 0.48 A. To prevent evaporation, during this process low viscosity silicone oil (approximately 5 ct) was placed around the geometry. The silicone oil should present no schematic error on rheometric data as this is below the rheometer’s torque range. Time sweep gelation measurements were conducted at a frequency and shear strain of 1 Hz and 0.5%, respectively. Gelation curves were fitted with an empirical function,
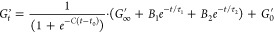
7where *C* and *t*_0_ are the rate and midpoint of increase of *G*′ due to photochemical cross-linking, respectively, *B*_1_ and *B*_2_ are the coefficients of the first and second relaxation mode, respectively, *G*_∞_^′^ is the plateau value of the storage modulus post-gelation and post-relaxation, and finally *G*_0_^′^ is the storage modulus of the sample pre-gelation. All other terms are defined within the main text. This function has previously been used to fit gelation curves of folded protein hydrogels assembled using this photochemical cross-linking method.^12^ Frequency sweeps were fit with a linear function to extract the relaxation exponent, *n*:
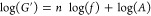
8where *f* is the fundamental oscillation frequency of measurement and *A* is a prefactor.

### Computational Modeling—BioNet

Our computational modeling utilized BioNet, a dynamic simulation platform designed to simulate network formation such as that observed within protein hydrogels.^56^ The specific implementations used in this work consisted of Brownian dynamics simulations of soft-core spheres (globular proteins) and chains of point-like particles connected by Hookean springs (unfolded proteins). The soft-core spheres and chains have specifically defined point-like sites representing cross-linking tyrosine residues, which interact with one another when within 3 Å by forming a permanent Hookean bond of length 1.5 Å, representing a carbon–carbon covalent cross-link. Further details not specific to this work can be found in a recent work by Hanson et al.^56^

We designed a system consisting of 5000 spheres, representing 100% folded protein, placed in a simulation box with periodic boundary conditions with a size defined to give a volume fraction of 0.074. Using this box, we created two systems, one with 91% (4550) spheres and another with 72% (3600) spheres. The remaining 9% and 28% were expanded into amino-acid chains: point-like particles connected by Hookean springs with properties defined to give the appropriate worm-like chain polymeric behavior of a disordered polymeric chain. We performed the simulation of each system three times using a statistically independent initial state each time. Statistical independence was achieved between initial states by first randomizing the initial positions of all particles, then allowing the system to relax under a steric potential in isolation, and finally performing a small amount of full Brownian dynamics (thermal noise, steric interaction, *etc*.) in the absence of any kinetic cross-linking. Simulations were run until sufficiently percolated; that is, 99% of all objects were connected to the biggest cluster in the box. All results quoted in the main work (fractal dimensions, elastic moduli, *etc*) were averaged over these three repeats to generate errors. These errors also include any fitting uncertainties carried forward (for example, from our box counting method).

Box counting for the simulations was equivalent to that performed previously, and the now present amino-acid chains were not accounted for.

## Supporting Information Available

CD and SAXS data demonstrating that monomeric BSA in solution in the absence or presence of DTT shows no significant change in the size or shape of the protein fold. Rheological data demonstrating the effects of adding DTT postphotochemical cross-linking. Additional SAS data and analysis. (PDF)
