# Supplementary material for: Control of Nanoscale In Situ Protein Unfolding Defines Network Architecture and Mechanics of Protein Hydrogels
Source: ACS Nano. 2021 Jul 2;15(7):11296–308. doi: 10.1021/acsnano.1c00353 (PMC8320229; doi:10.1021/acsnano.1c00353)
Supplement: Supplementary file 1 — nn1c00353_si_001.pdf [file nn1c00353_si_001.pdf]

Supporting Information

Control of Nanoscale *in situ* Protein Unfolding Defines Network Architecture and Mechanics of Protein Hydrogels

Matt D G Hughes[1], Benjamin S Hanson[1,2], Sophie Cussons[2,3], Najet Mahmoudi[4], David J Brockwell[2,3], Lorna Dougan*[1,2]

1 School of Physics and Astronomy, Faculty of Engineering and Physical Sciences, University of Leeds, Leeds, LS2 9JT, UK

2 Astbury Centre for Structural Molecular Biology, University of Leeds, Leeds, LS2 9JT, UK

3 School of Molecular and Cellular Biology, Faculty of Biological Sciences, University of Leeds, Leeds, LS2 9JT, UK

4 ISIS Neutron and Muon Spallation Source, STFC Rutherford Appleton Laboratory, Oxfordshire, OX11 0QX

Supplementary information

Data Access & Reproducibility

Simulations were performed using BioNet, a software package in development at the University of Leeds. This software is available for download from the Bitbucket repository https://bitbucket.org/GokuBH/proteinhydrogelsoftware/. Although the core software is in active development for additional applications and features, for reproducibility purposes the branch *BSAHydrogels* remains unchanged since this work was performed.

Simulations were performed using FFEA[70], a published software package available for download from the Bitbucket repository https://bitbucket.org/FFEA/ffea/src/master/. For reproducibility purposes the branch *BSAHydrogels,* which was slightly modified for this work, remains unchanged since this work was performed.

The core of both BioNet and FFEA is written in C++ for speed, and system initialisation and analysis tools are written in Python and utilise Numpy, Scipy and Matplotlib modules. Installation instructions can be found in the associated documentation and should be implemented on a Linux distribution.

A large portion of these simulations were performed with shared-memory parallelisation on the ARC3 supercomputing facilities at the University of Leeds.

All data and graphs used in this work, as well as additional movies which comprise the range of input parameterisations used in this work, can be found at https://doi.org/10.5518/940

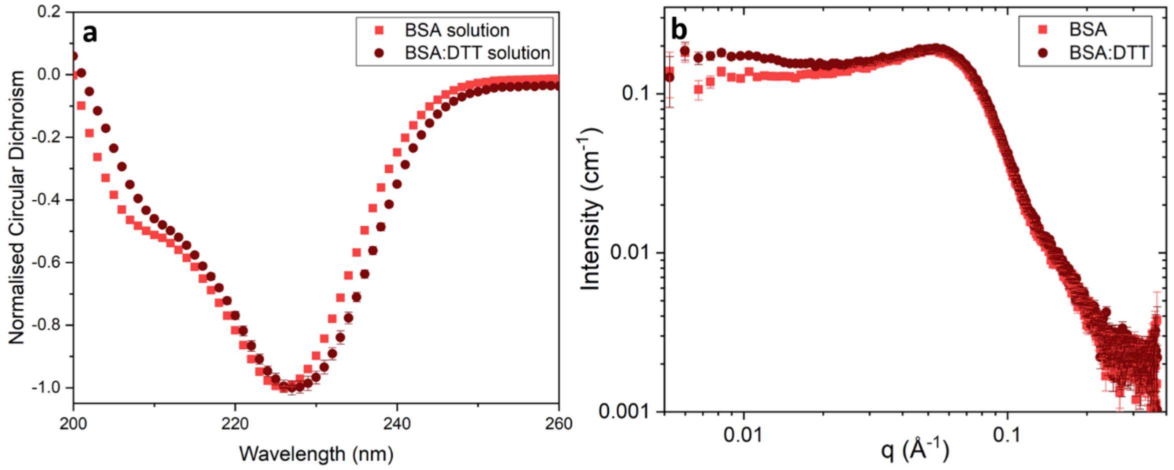

Figure S1: (a) Normalised CD spectra of BSA in solution (100mg/ml) in the absence and presence of DTT. (b) SAXS curves of 100mg/ml BSA solutions in the absence and presence of 3mM DTT.

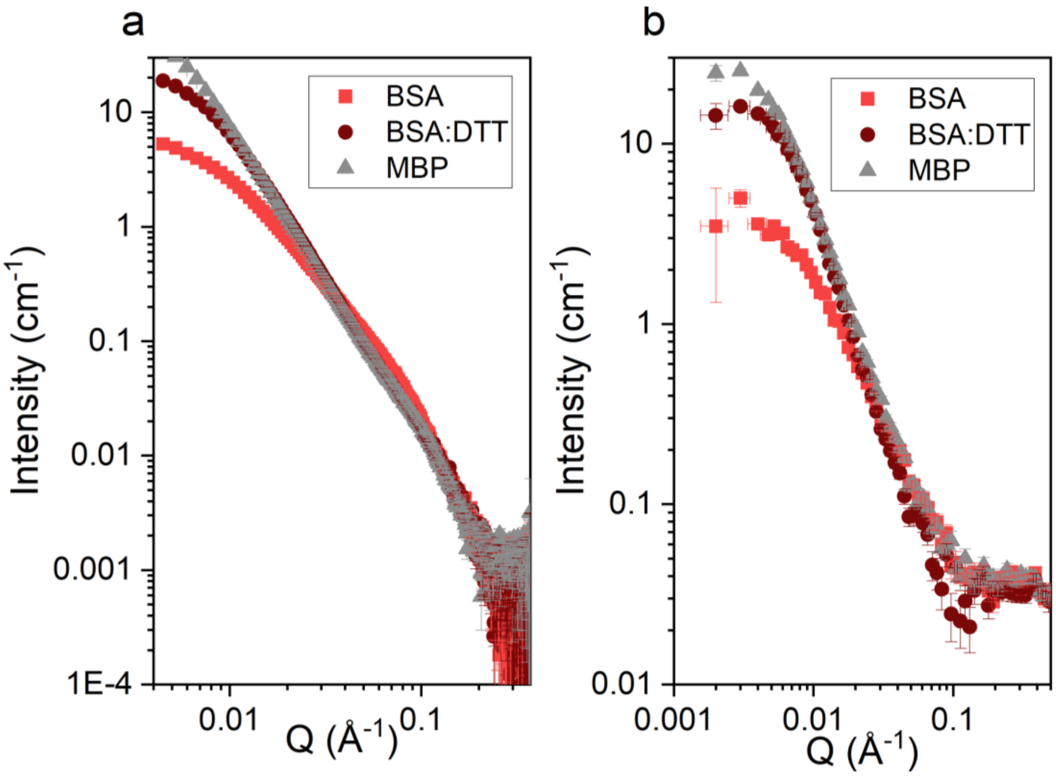

Figure S2: a) SAXS curves and b) SANS curves of folded BSA hydrogels (final concentrations: 100mg/ml BSA, 50mM NaPS, 100µM $Ru(BiPy)_3$) in the absence (light red) and presence (dark red) of DTT. Where previously published data on MBP[12] is included in grey.

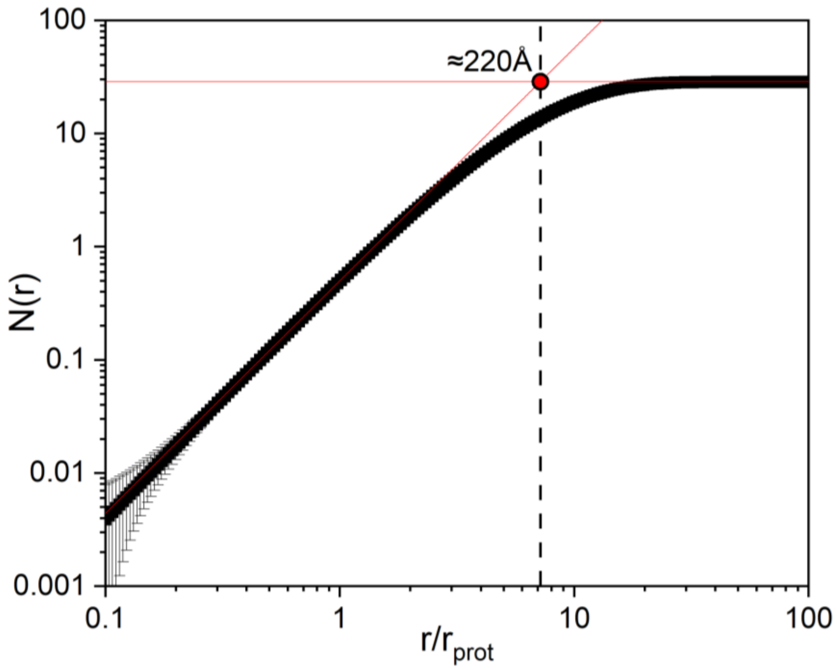

Figure S3: Exemplar plot of the number of protein monomers in a cluster as a function of distance from the centre of the cluster. Intersection of the red fitted lines (red dot) used to extract the number of proteins per cluster and estimate the cluster radius (dashed line).

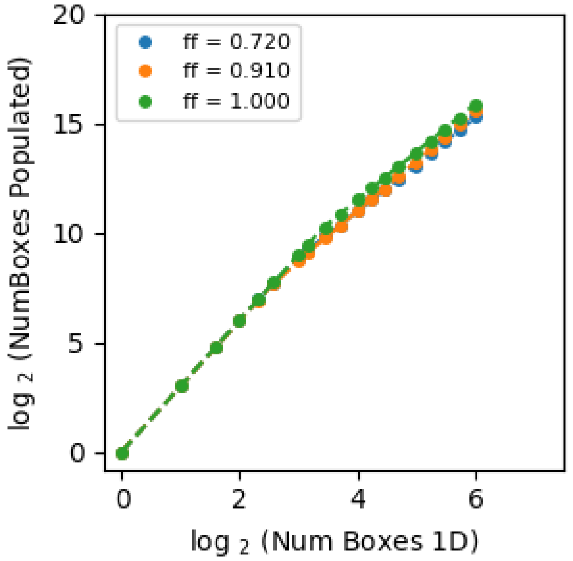

Figure S4: Number of boxes ‘filled’ as a function of total number of boxes, in the BioNet simulations with periodic boundary conditions. Using a two-segment piecewise fit, the first has gradient of three, the fractal dimension of cross-linked clusters in BioNet is given by the second gradient. “ff” is the folded-fraction.

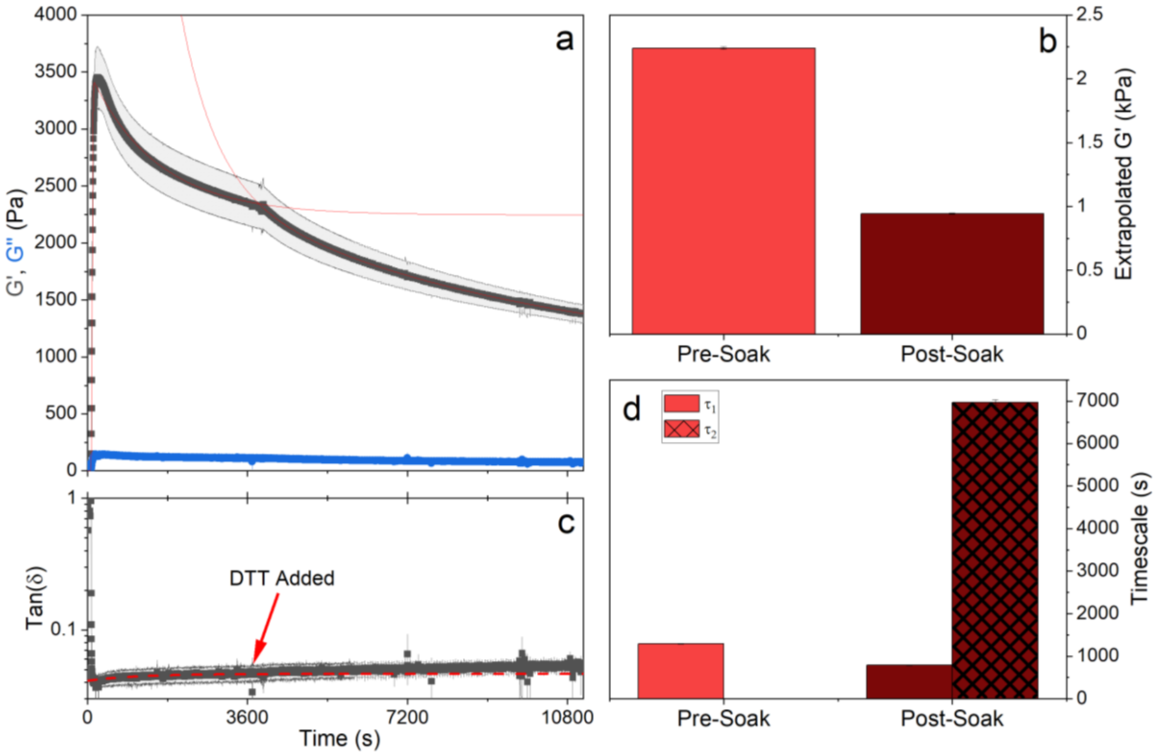

Figure S5: (a) Gelation curves, showing storage (closed symbols) and loss moduli (open symbols) *vs* time of a BSA hydrogel, where a DTT soak is added at t= 3960s. Red lines denote fits to extrapolate and extract the storage modulus and the relaxation timescales of the gel. (b) Extrapolated G’ values with and without the DTT soak extract from the fits in (a). (c) Loss ratio of a BSA hydrogel as a function of time, where DTT soak is added at t=3960s, denoted by the red arrow. (d) Timescales of relaxation extracted from the fits in (a), where the filed column is the

time constant of the network relaxation and the striped column is the time constant of the protein unfolding relaxation mode.

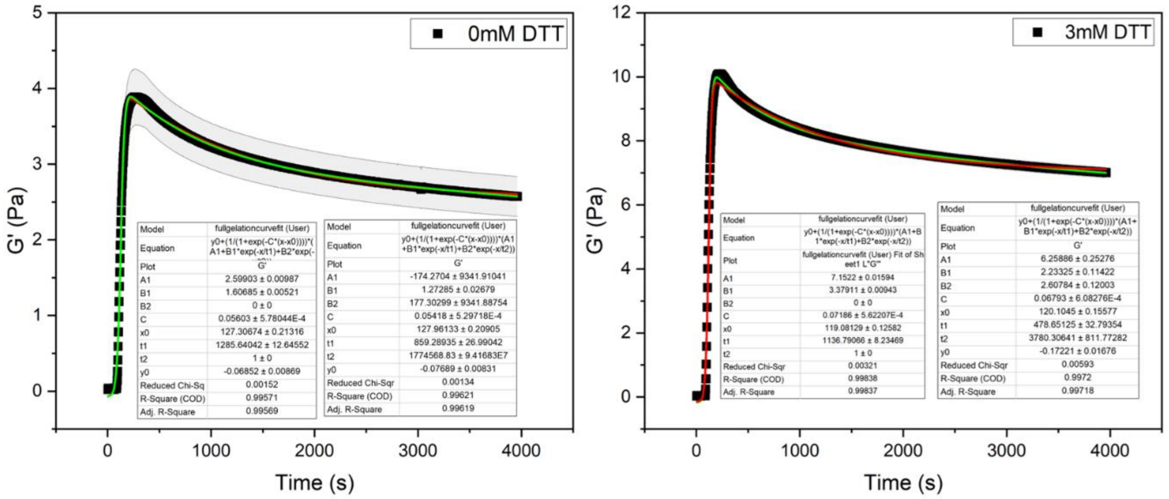

Figure S6: Gelation curves, showing storage (closed symbols) *vs* time of a BSA hydrogel in the absence (left) and presence (right) of DTT. The red lines shows fits where only one relaxation mode is included in the model (*i.e.* B2 is set to zero), and the green lines show the fit when two relaxation modes are included in the model. Tables of fit values are included to demonstrate non-sensical values obtained when fitting BSA in the absence of DTT with two relaxation mode model.
